# The Desirable Effects of Edoxaban on Thrombi in the Left Atrium Are Seemingly Connected to Pulmonary Vein Thrombi

**DOI:** 10.7759/cureus.52337

**Published:** 2024-01-15

**Authors:** Hidekazu Takeuchi

**Affiliations:** 1 Internal Medicine (Cardiology), Takeuchi Naika Clinic, Ogachi-Gun, JPN

**Keywords:** ischemic stroke, tee, 80-mdct, edoxaban, pulmonary vein thrombi, thrombi in the left atrium

## Abstract

Ischemic stroke (IS) causes various degrees of disability that sometimes induce social problems: therefore, preventing the occurrence and recurrence of IS is important. The recovery of motor function has been extensively studied. The characteristics of retrieved thrombi have become clearer; however, it is unclear what thrombi cause IS. Pulmonary vein thrombi (PVTs), such as those of the IS, can cause systemic thrombosis, by releasing several sizes of particles. PVTs are common but are underrated and can be detected via enhanced computed tomography (CT) and transesophageal echocardiography (TEE). PVTs often extend to the left atrium (LA) and can be mainly diagnosed using not enhanced CT but rather TEE. Extended LA thrombi are characterized by a lack of periodic movement with heartbeats. Direct oral anticoagulants (DOACs) are useful for preventing IS. We reported that rivaroxaban partially dissolved LA thrombi and PVTs using enhanced CT and TEE; however, the effects of edoxaban on LA thrombi and PVTs are unclear. We checked the images using enhanced CT and TEE and treated them with edoxaban. The patient was a 73-year-old female with easy fatigability. Edoxaban desirably affected the LA thrombi, and edoxaban partially dissolved the thrombi in the right lower pulmonary vein (RLPV). A decreased dose of edoxaban (15 mg, once a day) had similar effects on LA thrombi and RLPV thrombi, suggesting that this treatment could be useful for preventing IS.

## Introduction

Our previous study reported that at least 61% of elderly patients with chest pain had pulmonary vein thrombi (PVTs) [1], indicating that many elderly patients with age-related diseases have PVTs. We reported that the PVTs extended into the left atrium (LA) in some patients [1-6].

Ischemic stroke (IS) is a major cause of severe, persistent physical disability and the second leading cause of death worldwide. It is also the primary cause of work incapacity, and approximately 30-60% of all patients have some degree of physical disability after an acute event [7]. It is important to accurately define the mechanism of action of the IS to guide effective care and therapy. More than 15% of ISs are associated with cardiac embolism, and the most predisposed location is thought to be the left atrial appendage (LAA) [8]. In most cases, cardioembolism recurrence can be prevented by oral anticoagulants [9-11].

Therefore, for a patient with IS, early confirmation of a diagnosis of cardioembolic infarction is extremely important for initiating anticoagulation therapy for adequate secondary prevention. Moreover, the recovery of motor function after stroke has been a primary target of recent studies [12-15]. PVTs also have the potential to cause IS [16]; therefore, we need to pay attention to PVTs to prevent cardioembolism occurrence and recurrence.

Although PVTs are thought to be a rare complication of thoracic surgery or lung cancer, we have reported several cases of PVTs detected using 64-slice multidetector computed tomography (64-MDCT) [1-3,5], 80-slice multidetector computed tomography (80-MDCT) and transesophageal echocardiography (TEE) [4,6]. PVTs can occlude microvessels in all organs by releasing microclots. Occluded regions become hypoxic and undernourished, which may cause the organs to malfunction. It is important to determine how to treat LA thrombi and PVTs.

Direct oral anticoagulants (DOACs) are useful for preventing the occurrence and recurrence of IS [10,17-19]; however, the mechanisms underlying the effectiveness of DOACs are poorly understood. Using 80-MDCT and TEE, we reported that rivaroxaban partially dissolved LA thrombi and PVTs [6]; however, the effects of edoxaban on LA thrombi and PVTs are unclear. The present case report revealed the promising effects of edoxaban treatment on LA thrombi connected to the right upper pulmonary vein (RUPV) and the right lower pulmonary vein (RLPV), which could be assessed using not 80-MDCT but TEE. However, 80-MDCT could reveal that edoxaban treatment dissolved RLPV thrombi.

## Case presentation

A 73-year-old female with easy fatigability was assessed for heart function. She had symptoms of tachypnoea when she walked quickly. She had no symptoms of fever, cough, sputum, or IS. The respiratory exam did not reveal decreased breath sounds, lung crackles, or wheezing. The cardiac exam did not reveal a heart murmur or arrhythmia. The height was 165 cm, and the weight was 51.7 kg. A chest roentgenogram revealed no lung cancer or cardiomegaly. No previous treatment with warfarin had been performed. ECG indicated sinus rhythm, a normal axis, and no ST-T changes, and the patient’s heart rate was 73 beats/min. The serum D-dimer level was 0.7 μg/mL (normal; < 1.0 μg/mL), the activity of protein S was 112% (normal; 74-132%), and the activity of protein C was 124% (normal; 64-135%). The homocysteine level was 8.9 nmol/mL (normal; 5-15 nmol/mL). Her brain natriuretic peptide (BNP) level was 56 pg/mL (normal; 0.0-18.4 pg/mL). The C-reactive protein (CRP) level was 0.30 mg/dL (normal; 0.00~0.19 mg/dL). To scrutinize the thrombi in the LA and pulmonary veins, TEE and 80-MDCT were performed.

**Figure 1:**
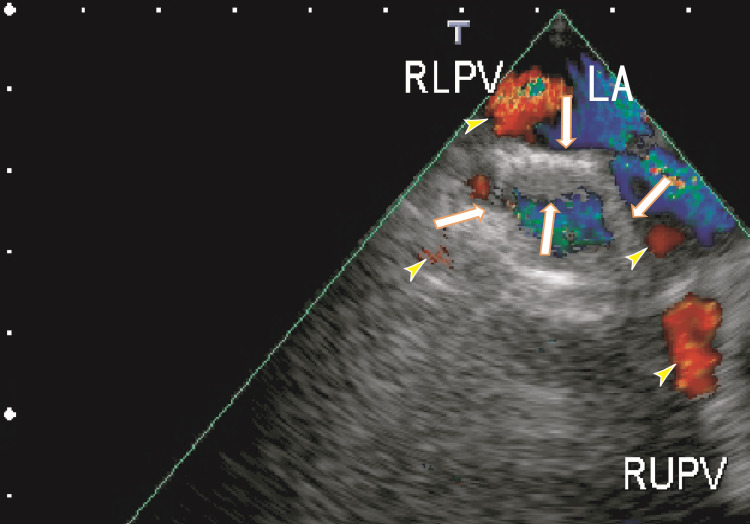
TEE images depicting thrombi in the left atrium before treatment TEE images depicting thrombi in the left atrium (LA) as dog-like shapes that appeared to be connected to the entrance of the right upper pulmonary vein (RUPV) and the right lower pulmonary vein (RLPV) (arrows). The thrombi included a large white area and were linear in shape. The blood flows from the RUPV and RLPV are shown as red areas (arrowheads). LA, left atrium; RLPV, right lower pulmonary vein; RUPV, right upper pulmonary vein

**Figure 2:**
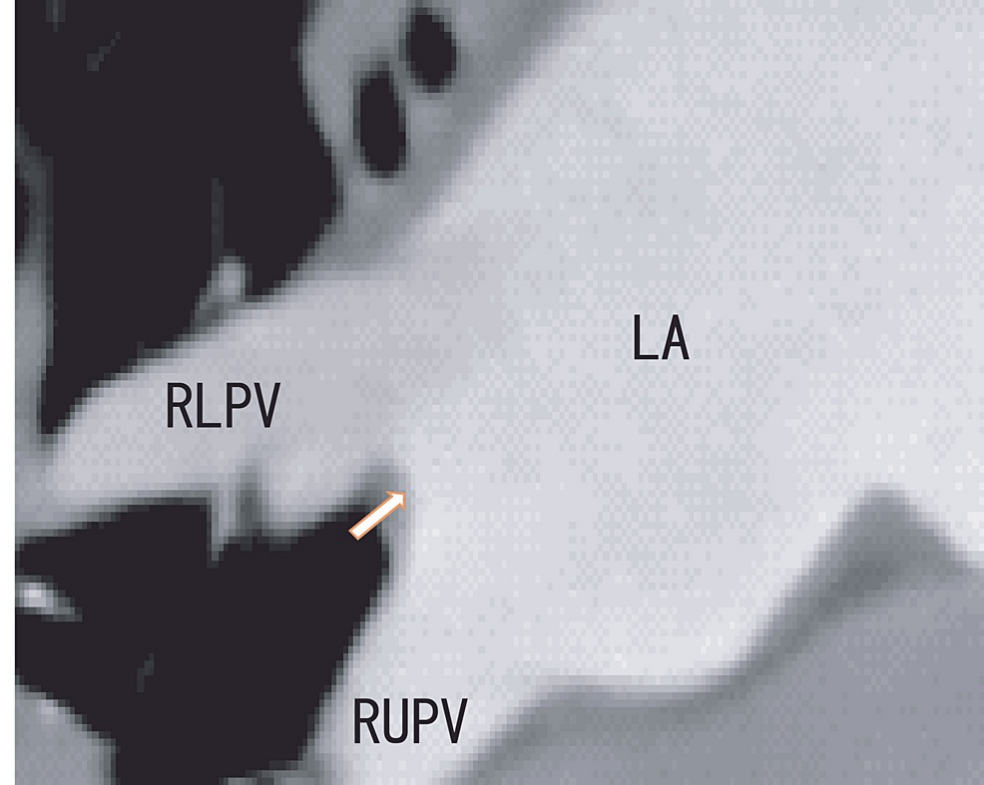
Oblique images from an 80-MDCT scan revealing the RUPV, RLPV, and LA before treatment Oblique images from an 80-MDCT scan revealing the RUPV, RLPV, and LA; the location was similar to that in the TEE images in Figure 1. There were no images of thrombi in the left atrium (arrow). Additionally, there was a rather dark area in the RLPV. LA, left atrium; RLPV, right lower pulmonary vein; RUPV, right upper pulmonary vein

TEE demonstrated thrombi in the LA as dog-like shapes. The thrombi were connected to the entrance of the RUPV and RLPV. The LA thrombi had no periodical movement with heartbeats (Fig. 1 and Video 1). 80-MDCT demonstrated no thrombi in the LA; however, the RLPV appeared darker, indicating thrombi in the RLPV (Fig. 2).

**Video 1:** TEE images revealing thrombi in the left atrium TEE images revealing thrombi in the left atrium (LA) that appeared to be connected to the entrance of the right upper pulmonary vein (RUPV) and right lower pulmonary vein (RLPV). The thrombi, including the large white area, were linear in shape and did not clearly move inward. The blood flows from the RUPV and the RLPV are shown as red areas.

**Figure 3:**
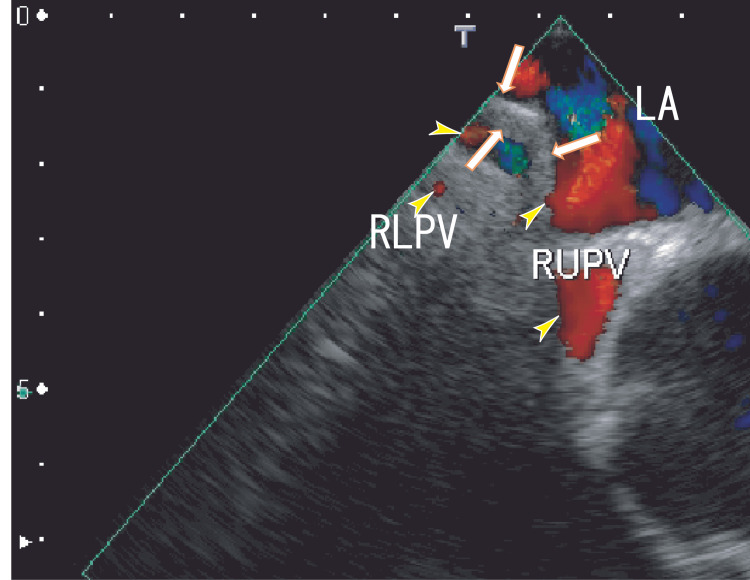
TEE images obtained after six months of edoxaban treatment TEE images obtained after six months of edoxaban treatment revealing thrombi in the LA that appeared to be connected to the entrance of the RUPV (arrows). The shape of the thrombi, including some white areas, was curved. The blood flow in the RUPV and RLPV are shown as red areas (arrowheads). LA, left atrium; RLPV, right lower pulmonary vein; RUPV, right upper pulmonary vein

**Figure 4:**
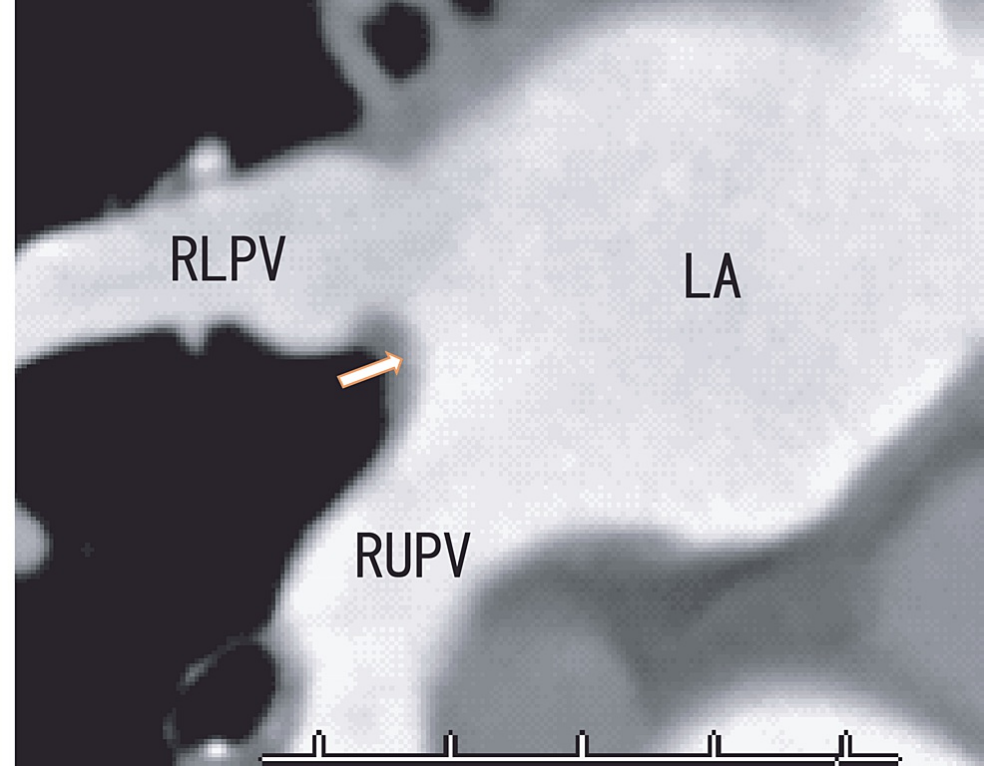
Oblique images from an 80-MDCT scan obtained after six months of edoxaban treatment Oblique images from an 80-MDCT scan obtained after six months of edoxaban treatment revealing the RUPV, RLPV, and LA; the angle was similar to that of the TEE images in Figure 3. There were no images of thrombi in the LA (arrow). The dark area of the RLPV became slightly clearer, indicating that the thrombi had partly resolved. LA, left atrium; RLPV, right lower pulmonary vein; RUPV, right upper pulmonary vein

After six months of edoxaban treatment (30 mg once a day), the thrombi appeared in a similar position as before treatment. However, the hyperechoic area of the thrombi decreased, and the shape of the thrombi showed curved areas (Fig. 3 and Video 2). 80-MDCT demonstrated no thrombi in the LA; however, the RLPV appeared clearer than previously observed (Fig. 4). Her BNP level was 49 pg/mL, and her CRP level was 0.02 mg/dL.

**Video 2:** TEE images obtained after six months of edoxaban treatment TEE images obtained after six months of edoxaban treatment revealing thrombi in the LA that appeared to be connected to the entrance of the right upper pulmonary vein (RUPV) and right lower pulmonary vein (RLPV). The thrombi, including some white areas, were curved and did not move extensively. The blood flows from the RUPV and the RLPV are shown as red areas. The thrombi moved more toward each other than in Video 1.

**Figure 5:**
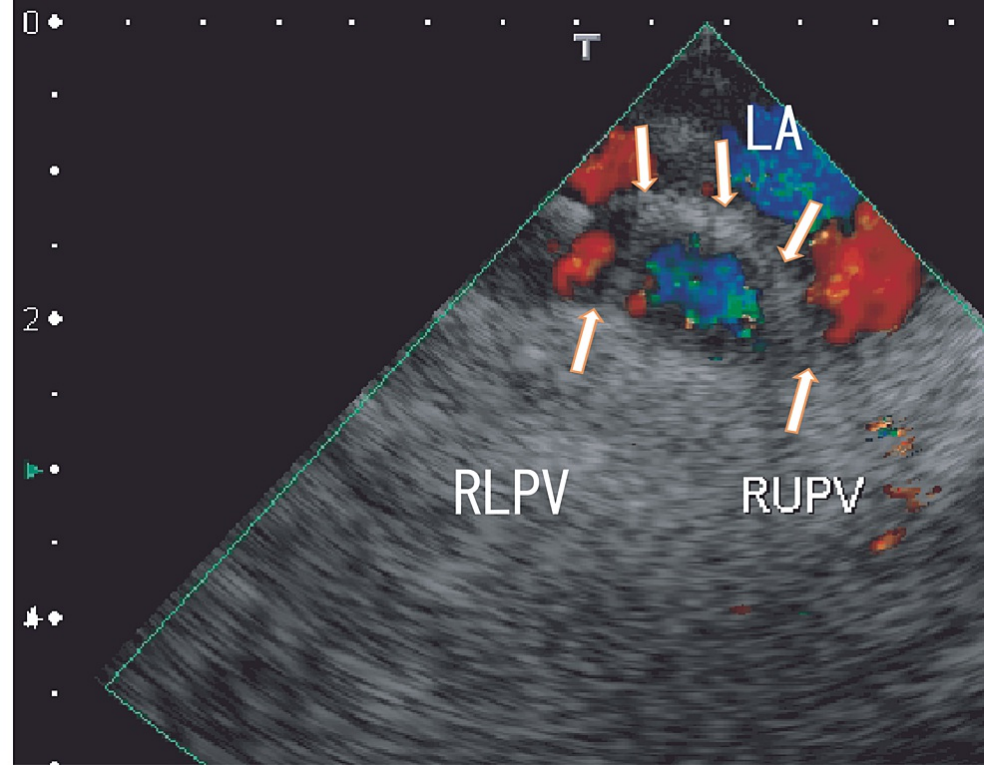
TEE images obtained after 18 months of edoxaban treatment TEE images obtained after eighteen months of edoxaban treatment revealing thrombi in the LA that appeared to be connected to thrombi in the RUPV and RLPV (arrows). The shape of the thrombi, including the small, rather white area, was curved. The thrombi connected to the RUPV seemed to become broader. LA, left atrium; RLPV, right lower pulmonary vein; RUPV, right upper pulmonary vein

**Figure 6:**
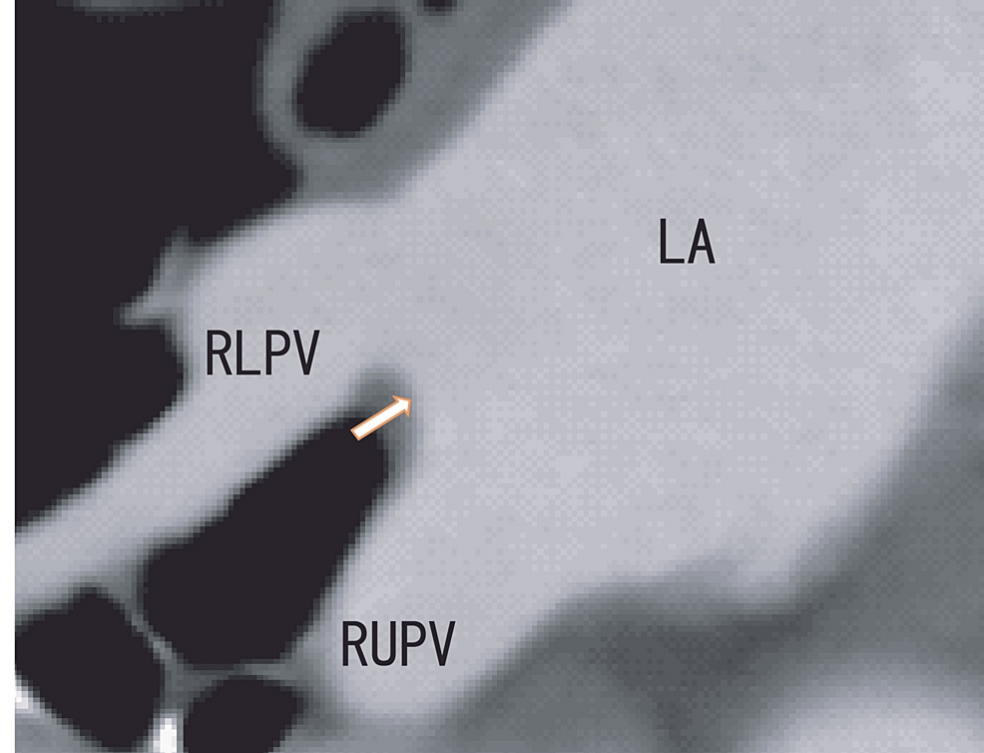
Oblique images from an 80-MDCT scan obtained 18 months of edoxaban treatment Oblique images from an 80-MDCT scan obtained eighteen months of edoxaban treatment showing the RUPV, RLPV, and LA; the location was similar to that of the TEE images in Figure 5. There were no images of thrombi in the LA (arrow). The dark area of the RLPV became clearer, indicating that the thrombi had resolved further. LA, left atrium; RLPV, right lower pulmonary vein; RUPV, right upper pulmonary vein

After an additional 12 months of edoxaban treatment (15 mg once a day), the thrombi appeared in a similar position as previously described. However, the hyperechoic area of the thrombi decreased and became much darker, and the thrombi were curved (Fig. 5) and moved periodically with increasing heartbeat (Video 3). 80-MDCT demonstrated no thrombi in the LA, and the RLPV appeared clearer than previously observed (Fig. 6). Her BNP level was 48 pg/mL, and her CRP level was 0.01 mg/dL. During edoxaban treatment, the patient showed no symptoms of IS or any bleeding.

**Video 3:** TEE images obtained after 18 months of edoxaban treatment TEE images obtained after eighteen months of edoxaban treatment revealing thrombi in the LA that appeared to be connected to the entrance of the right upper pulmonary vein (RUPV) and right lower pulmonary vein (RLPV). The thrombi, including the small white area, were curved and periodically moved inward. The blood flows from the RUPV and the RLPV are shown as red areas.

## Discussion

To our knowledge, the present case report is the first to show that edoxaban affects LA thrombi through the entrance of both the RLPV and the RUPV; however, these effects could be assessed using TEE rather than 80-MDCT. The thrombi did not move like the LA wall at first (Video 1); therefore, LA thrombi may connect RUPV thrombi and RLPV thrombi because PVTs do not move periodically with heartbeats in many cases [6]. Importantly, 80-MDCT images sometimes cannot reveal LA thrombi [6].

Edoxaban partially dissolved thrombi in the RLPV, suggesting that edoxaban may prevent IS by decreasing these thrombi. According to our previous case report, rivaroxaban partially dissolved LA thrombi [6]. Edoxaban did not dissolve thrombi in the LA but did affect LA thrombi. After edoxaban treatment, the LA thrombi lost some of their white areas, became darker, and became flexible enough to curve. The thrombi did not move inward at first (Video 1), but after treatment, the thrombi moved inward periodically with the heartbeats (Videod 2-3). The mechanisms underlying the changes in LA thrombus movement are unclear. However, the change in the LA thrombus density after edoxaban treatment indicated that edoxaban has the potential to resolve LA thrombi in the end and that the features are in fact thrombi. When rivaroxaban partially dissolved the LA thrombi, the thrombi connected to the RLPV thrombi and extended along the posterior wall of the LA; however, the present LA thrombi existed near the entrance of the RUPV, which indicated that the situation was near the anterior wall of the LA, and the LA thrombi were rather whitish. The difference in the response of LA thrombi may depend not on the medication used but on the traits of the LA thrombi. However, additional studies are needed to clarify these issues.

Additionally, according to the results of the present study, decreased edoxaban treatment (15 mg once a day) may ameliorate both RLPV and LA thrombi. In particular, the thrombi in the RLPV seemed to disappear, as estimated using 80-MDCT. Decreased edoxaban treatment (15 mg once a day) may be useful for preventing IS.

Edoxaban treatment might ameliorate CRP levels from 0.30 mg/dL to 0.01 mg/dL, indicating that edoxaban affects systemic inflammation [20]. Edoxaban may ameliorate inflammation by inhibiting the release of microclots that occlude microvessels in all organs. However, additional studies are needed to clarify these issues. The level of BNP decreased only slightly.

## Conclusions

There were dog-like thrombi in the LA, which were observed via TEE rather than 80-MDCT. First, the thrombi did not move simultaneously with the heartbeats and were linear in shape. After six months of edoxaban (30 mg, once a day) treatment, the LA thrombi became darker and partly moved simultaneously with the heartbeats.

After an additional 12 months of edoxaban (15 mg, once a day) treatment, the LA thrombi became much darker and moved simultaneously inward with heartbeats. It is possible that a decreased dose of edoxaban (15 mg, once a day) may be useful for preventing IS.
